# Synthesis and Characterization of Layered Double Hydroxides as Materials for Electrocatalytic Applications

**DOI:** 10.3390/nano11030725

**Published:** 2021-03-13

**Authors:** Domenica Tonelli, Isacco Gualandi, Elisa Musella, Erika Scavetta

**Affiliations:** Department of Industrial Chemistry “Toso Montanari”, University of Bologna, Viale Risorgimento 4, 40136 Bologna, Italy; isacco.gualandi2@unibo.it (I.G.); elisa.musella3@unibo.it (E.M.); erika.scavetta2@unibo.it (E.S.)

**Keywords:** layered double hydroxides, electrocatalysis, syntheses, characterization

## Abstract

Layered double hydroxides (LDHs) are anionic clays which have found applications in a wide range of fields, including electrochemistry. In such a case, to display good performances they should possess electrical conductivity which can be ensured by the presence of metals able to give reversible redox reactions in a proper potential window. The metal centers can act as redox mediators to catalyze reactions for which the required overpotential is too high, and this is a key aspect for the development of processes and devices where the control of charge transfer reactions plays an important role. In order to act as redox mediator, a material can be present in solution or supported on a conductive support. The most commonly used methods to synthesize LDHs, referring both to bulk synthesis and in situ growth methods, which allow for the direct modification of conductive supports, are here summarized. In addition, the most widely used techniques to characterize the LDHs structure and morphology are also reported, since their electrochemical performance is strictly related to these features. Finally, some electrocatalytic applications of LDHs, when synthesized as nanomaterials, are discussed considering those related to sensing, oxygen evolution reaction, and other energy issues.

## 1. Introduction

Layered double hydroxides (LDHs) are lamellar compounds having molecular formula [M(II)_1−x_M(III)_x_(OH)_2_]^x+^(A^n−^_x/n_)·mH_2_O, where x ranges from 0.22 to 0.33, M is a metal and A^n−^ is a n− valent anion. They are also called hydrotalcite like compounds as LDHs are synthetic materials coming from the natural hydrotalcite, i.e., Mg_2_AlOH(CO_3_). This clay consists of positively charged brucite-like layers (brucite = Mg(OH)_2_) where the cations are octahedrally coordinated with OH^−^, and of interlayer anions balancing the positive charge due to the partial substitution of the bivalent Mg with the trivalent Al. The peculiar property of LDHs is the possibility to exchange the interlayer anions; for this reason, they are also named anionic clays. These clays are employed in many fields such as medicine (especially in drug delivery and release), environment (to remediate pollution), biotechnology, as precursors for catalysts, and in electrochemical applications (electrocatalysts, sensors, oxygen evolution reaction (OER), energy storage, fuel cells, etc.) [1].

In the context of the last mentioned applications, it is essential to employ conductive LDHs, and that is possible when transition metals (such as Co and Ni) are present in the brucitic layers. In such a case, an inner reaction involving the redox active metal centers of the clay occurs within a proper potential window. The charge transport occurs throughout the material with a mechanism which is based on the electron hopping and anions movement (from the solution inside the clay during oxidation, and vice versa during reduction, similarly to the one typical of conducting polymers). This mixed conduction mechanism is favored by alkaline media [2].

In the past few years, nanomaterials have attracted much attention due to their peculiar physicochemical properties differing significantly from those displayed by the same bulk materials [3]. For example, nanomaterials possess exceptional electrical and catalytic properties, large surface-to-volume ratio (S/V, aspect ratio), and a large number of adsorption-active sites which make them particularly suitable for electrocatalytic applications. Electrocatalysis is the field of chemistry that deals with the catalysis of redox reactions, and it is a key phenomenon for electrochemical processes and devices in which the control of interfacial charge transfer reactions plays an important role. In order to act as a redox mediator, a material can be present in solution or supported on a conductive substrate. In the latter case, there are many advantages in terms of the small amount of requested materials, higher concentration of the active centers, and therefore greater efficiency for the redox reaction to take place [4]. Obviously, when the redox mediator is someway anchored onto a support, another key property is the adhesion between the two phases, which must assure the formation of a mechanically stable coating. The methods to modify a conductive surface can involve adsorption, covalent bond formation, coating with previously synthesized materials, e.g., soluble polymers, or electrodeposition, when the modifiers can be electrosynthesized [2].

The properties of nanomaterials strongly depend on their size, morphology, and shape, which in turn are related to the used synthetic procedure [5]. LDHs can be synthesized with a lot of procedures. The most commonly used is the chemical route, which involves bulk synthesis, but also other synthetic approaches are available, among which the electrochemical deposition. This strategy ensures the obtainment of LDH films on any kind of conductive supports of any shape and dimension, including porous substrates and transparent or flexible electrodes.

As stated above, when redox-active metals are present in the brucitic layers, i.e., a reversible electrochemical process involving these cationic sites can occur within an appropriate potential range, LDHs materials can act as redox mediators to electrocatalyze the oxidation of many compounds.

The aim of this paper is to summarize the most commonly used methods to synthesize and characterize LDHs and to present some of the most outstanding electrocatalytic applications of LDHs, when synthesized as nanomaterials, considering in particular those related to sensing, OER, and in general to the energy issues.

## 2. Preparation of the Devices

The preparation of the devices, as already stated, can be basically accomplished through two approaches:

(i) the chemical bulk synthesis of the LDH and subsequent electrode coating;
(ii) the direct synthesis of LDH on the electrode surface.

The first procedure has the advantage that the LDH, being produced in bulk in a large amount, can be easily characterized, allowing a fine control of its morphology and composition through well-known and consolidated techniques. On the other hand, the subsequent step of electrode modification is often critical, because it is hard to obtain a good adhesion and mechanical stability of the LDH coating. The electrodeposition of LDHs, which is a peculiar example of direct synthesis, has the main advantage of ensuring a better adhesion of the material to the support, and allowing for an easier control of the thickness of the LDH layer. The major disadvantage is that, being the LDH generally produced in very low amounts, its characterization is much more difficult; moreover, most of the proposed methods do not permit a fine control of the LDH composition and morphology. In the following, the most commonly employed procedures to synthesize LDHs for electrocatalytic applications will be discussed and compared with the aim to highlight their advantages and disadvantages and to correlate the effect of the device preparation on its performance.

### 2.1. LDH Bulk Synthesis

The typical procedures to perform the bulk synthesis are based on the co-precipitation, the ion-exchange, the calcination recovery, the hydrothermal synthesis, the microemulsion method, and the layer-by-layer deposition.

Among them, the most commonly employed method is the co-precipitation in low oversaturation conditions [6], which is based on the dropwise slow addition of a base (usually NaOH) to an aqueous solution, containing the metals at a proper molar ratio and concentration, kept under stirring. The pH is controlled during the whole synthesis to a target value, the precipitate is aged in contact with the mother solution until it is filtered, washed, and dried.

The ion-exchange method starts from an already synthesized LDH containing an easily exchangeable interlayer anion [1,7], and consists in the replacement of the original anion with a new guest species to obtain a different LDH. A drawback of such a procedure is that the exchange reaction sometimes is not complete, and consequently the method exhibits a low efficiency.

Both the co-precipitation and the ion exchange methods can be employed to prepare LDHs of variable composition, using different combinations of bivalent and trivalent cations, but often lead to poorly crystalline materials; in order to improve crystallinity a thermal ageing step can be conducted after the co-precipitation or ion-exchange occurrence [8].

Another well-known method for LDHs synthesis is calcination recovery which is based on the “memory effect” [9]. The interlayer anion is removed throughout a calcination process that converts the LDH into a layered double oxide (LDO) with the layered structure collapsing to a cubic phase. When the LDO is added to an aqueous solution the layered structure is rebuilt again, with a different intercalated anion, thus obtaining another LDH.

More recently, other synthesis methods have been proposed to improve LDH crystallinity and to obtain LDHs with a more uniform and controlled morphology. For example, using urea as the hydrolysis agent, LDHs with large, thin platelets and narrow particle size distributions can be easily obtained. These characteristics are achievable by co-precipitation only after extensive ageing. The method is mainly restricted to Al-based LDHs, but more recently tri-metal LDHs with composition Mg/M/Al (with M = Fe, Co, Ni, Cu or Zn) have been synthesized, where Mg is partly substituted with another bivalent cation, but even Al can be substituted by Fe [10]. The urea hydrolysis method has been further improved by adding sodium citrate as a chelating reagent to the synthesis solution, and this procedure has been successfully employed to obtain Ni/Fe LDHs with high crystallinity and well-defined hexagonal-shaped crystallites [11].

Another preparation procedure, which allows for the synthesis of micrometer scale LDH particles, is the hydrothermal method which consists in heating a mixed homogeneous slurry of a bivalent and a trivalent metal (usually in the form of oxides or nitrate salts) to obtain the corresponding LDH. The morphology and the size of the LDH particles can be varied by controlling the heating temperatures and the metals ratio. According to this procedure, the formation of Mg/Al LDH has been extensively investigated [12], but, more recently, composites based on Ni/Fe LDHs and carbon nanotubes (CNTs) or reduce graphene oxide (RGO) have been prepared and characterized for the development of supercapacitors [13].

One of the main drawbacks of physical deposition methods is that the adhesion between LDH crystallites and the underlying conductor is weak, and sometimes some detachments can occur, especially when the electrode is polarized. An improvement in film adhesion can be obtained by decreasing the particles but this is a challenging topic. Several strategies have been implemented to this aim when performing the synthesis by co-precipitation.

A fine control of co-precipitation conditions can narrow the particles size down to 40–300 nm [14], and separating the nucleation and ageing steps can further adjust the sizes in a narrower region (60–80 nm) [8]. Another strategy involves the vigorous stirring of the mixed solution of metal nitrates and sodium hydroxide in methanol medium to form a LDH slurry, which evolves into LDH nanoparticles (∼40 nm) after a further solvothermal treatment [15].

LDHs can be also synthesized by a sol gel like process that is based on the hydrolysis and condensation of alkoxide precursors in alcohol [16] or on the hydrolysis of acetate precursors in polyol medium [17]. This method has several advantages over other synthetic procedures. It is a simple way to obtain nanoscaled particles with high specific surface area and narrow pore size distribution and allows for an accurate control over structural and textural properties of the products which are obtained with high-purity. Several LDHs have been successfully synthesized by the sol gel procedure, e.g., those containing Mg/Al [18,19], Mg/Ga [19], Mg/In [19], Ni/Al [18], and Zn/Al [20].

Another promising synthesis that enables the dimensions of LDHs particles to be decreased to a nanometer level is the microemulsion method, where the synthesis is carried out in a solution typically composed of oil, water, and a surfactant (occasionally with a co-surfactant). The process exploits the confined environment of the water pools of reverse microemulsions that can be considered as nanosized reaction chambers, and thus constitutes an efficient tool to control the nucleation and growth of inorganic precipitates. Recently, O’Hare and Davis’ group [21] prepared Mg/Al LDH nanocrystals in a water-in-oil microemulsion of the anionic surfactant sodium dodecyl sulfate, isooctane, and water. More recently, the production of several LDHs (Ni/Al, Zn/Al, Ni/Cr, Zn/Cr, Ni/Fe and Mg/Fe) by the double microemulsion technique with a cationic surfactant has been reported [22,23]. Bellezza et al. employed a quaternary water-in-oil microemulsion of cetyltrimethylammonium bromide/n-butanol/isooctane/water, and obtained LDH nanoparticles whose mean size was in the 10–30 nm range [22].

For electrochemical applications, whichever the procedure employed for the synthesis, the LDH must be deposited on a conductive surface. LDH-modified electrodes are usually prepared by coating the surface of the working electrode (metal or carbon based) by a film of controlled thickness [24].

Typically, the coating is obtained by depositing a fixed amount of a colloidal suspension of a chemically synthesized LDH, sometimes with the help of a spin-coater, and allowing it to dry in air. To improve the mechanical adhesion a binder (polytetrafluoroethylene, acetylene black, or Nafion) is often added.

In order to obtain LDH thin films the layer-by-layer (LbL) deposition can be employed, which exploits the electrostatic self-assembly technique. Sasaki and co-workers have deeply studied this procedure [25] consisting of the delamination of LDHs into single positively charged nanosheets that are then alternated to negatively charged species and assembled on the electrode surface [26].

The adherence of the film to the electrode substrate can be also improved by means of methods that are based on the one step synthesis of the LDH directly on the conductive substrate (in situ growth methods). These procedures are very rapid and allow, at the same time, for the synthesis of the LDH and for the electrode modification, and they are not limited by the shape of the substrate. The main procedures developed for the realization of electrode coatings to be employed in the fields of electrocatalysis are described and compared in the following paragraph.

### 2.2. In Situ Growth Methods

One of the most interesting in situ growth methods to realize the one step modification of a conductive surface is the electrochemical deposition. This procedure, firstly reported by Kamath’s group [27] to synthesize LDHs containing Co(II) or Ni(II) and Al(III), is based on the cathodic reduction of nitrate ions in a solution containing a bivalent and a trivalent metal in order to generate a basic solution that allows the precipitation of the LDH. Starting from those results, Tonelli’s group conducted an extensive study aimed at finding the best experimental conditions to modify several electrode materials by electrosynthesis of a Ni/Al-NO_3_ LDH [28,29]. The initially-adopted procedure was based on a potentiostatic deposition, where a cathodic potential was applied to the working electrode for fixed time lengths; the pulse length (usually between 30 and 120 s) determined the thickness of the LDH coating that ranged between 100 and 800 nm. The procedure was applied to synthesize LDHs having Al as the trivalent metal and Ni or Co as the bivalent one, and later the substitution of Fe with Al was taken into account to increase the conductivity properties of the LDHs [4,30,31]. The electrochemical deposition also allowed the synthesis of LDHs with a more complex composition (containing Mg(II) and variable amounts of Rh(III)) on FeCrAlY alloy foams to be used as precursors of Rh-based catalysts for the catalytic partial oxidation of methane [32]. Recently, an alternative synthesis protocol based on a potentiodynamic deposition has been studied and optimized to better and finely control the composition of the LDH materials. In particular, one of the main issues of the potentiostatic or galvanostatic deposition lies in the possibility to control the ratio Me(II)/Me(III) of the LDH. In fact, the ratio of the two metals in the electrodeposited compound is not strictly the same as present in the electrolytic solution because of the concentration gradients originating during the synthesis, especially when a long potential pulse is applied. Our group [33,34,35] has demonstrated that the potentiodynamic approach, which is based on the application of few CV cycles in the cathodic potential range, provides a reproducibility of the deposit which is much better than the one achieved with either the galvanostatic or potentiostatic methods. This is due to the strong reduction of cations concentration gradients in the diffusion layer, which are typical of the potentiostatic approach, thus allowing the restoration of the initial concentrations at the electrode surface. The result is that the LDH Me(II)/Me(III) ratio is the same as the one present into the electrolytic bath.

The advantages of the electrochemical deposition are the short time needed to obtain the LDH film, the possibility of coating complex geometry supports and to easily modulate both the electrolytic bath composition and the electrosynthesis time to tune the film thickness and the M(II)/Me(III) molar ratios. One drawback is the low crystallinity exhibited by the LDH which is due to the short time of the synthesis so that the material does not have time to reorganize its structure. Moreover, the electrochemical deposition does not allow the orientation of the LDH material and the morphology of the film to be finely controlled.

Recently, some authors have proposed one step methods to directly synthesize more oriented LDHs with the aim to enhance their specific surface area and increase their conductivity. One of the most interesting procedures has been proposed by Chang Yu and coworkers [36] who reported a simple and efficient strategy for assembling vertically oriented Ni/Co-LDH nanoarrays (Ni/Co-LDH-NA) on carbon fiber papers (CFP) to be employed for OER in water splitting. The CFP, being a current collector and a robust substrate, is capable both of promoting fast electron transport and to modulate the LDH assembly. Briefly, the procedure consists of the addition of cobalt and nickel nitrates, and of cetyltrimethylammonium bromide to a solution of ethanol and water containing the CFP substrate, followed by ultrasonication for 30 min, and a hydrothermal treatment at 180 °C for 12 h.

LDHs with a vertical orientation have been also synthesized by Qian Xiang and coworkers [37] who prepared Fe/Ni LDH nanosheet arrays on various metal foils by a facile hydrothermal method. The synthesis consisted of the precipitation of iron and nickel on the metal foil in aqueous solution, which was induced by urea hydrolysis, in alkaline solution, upon hydrothermal treatment at 120 °C for 12 h. The LDH grows in a vertical direction with respect to the metal foil because CO_3_^2−^ anions, produced by urea decomposition, adsorb on the (001) surface and passivate this plane so that to suppress the LDH growth along this direction, and causing the stacking of the crystals in the direction vertical to the substrate surface.

Vertically grown LDH nanosheet arrays are particularly interesting, because on one hand many nanoscale channels are created, thus enabling an easy access for the reaction intermediates to the catalytic active sites, and on the other hand they ensure the direct contact of each individual sheet with the conductive substrates, thus promoting the intralayer electron transfer.

## 3. Characterization

LDHs are usually obtained from the above-mentioned chemical strategies as microcrystalline powder and, as such, invariably contain defects which can thoroughly influence their physical and chemical properties. Powder X-ray diffraction (PXRD) is certainly the most widely used technique to study the structure and microstructure of crystalline solids like LDHs. The microstructure can strongly affect the diffraction line profiles, leading to broadened and occasionally shifted or asymmetric reflections. Nevertheless, LDH patterns show some general features that can be considered peculiar of all LDH phases, i.e., the presence of sharp and intense lines at low values of the 2θ angle, and of less intense and generally asymmetric lines at higher angular values. Summarizing, the reflections in the XRD pattern of a typical LDH fall into three groups. Firstly, a series of strong basal (00l) reflections at low angles allow the direct determination of the basal spacing normal to the (00l) plane (c_o_), which is equal to the thickness of one brucite-like layer plus one interlayer. Secondly [38], the position of the (110) reflection at high angle (near 60° 2θ for Cu Kα radiation) allows the value of the lattice parameter a_o_ to be determined, since a_o_ = 2d_(110)_. The value of a_o_ corresponds to the distance between two metal cations, and it should, therefore, reflect the radii of the cations. Finally, the positions of the (01l) and/or (10l) reflections at intermediate angles can be used to determine the stacking pattern of the layers. There are a number of reasons why the XRD peaks of LDH samples are often rather broad. The relatively small domain size, particularly in the (00l) direction, leads to line broadening. The Scherrer equation may be used to estimate the domain size in the a and c directions from the width of the (110) and (00l) reflections, respectively, although the inherent approximations to this method should always be highlighted [1,39,40]. In particular, when nanosized systems are involved, XRD might not be the best choice, so alternative (or at least complementary) approaches are needed.

IR and Raman analyses are not considered diagnostic tools for LDHs, but they are widely employed to identify the presence of foreign anions in the interlayer among the brucite-like sheets as well as to gather information about the bonds formed by the anions, and about their orientations [1,41,42,43,44]. Consequently, these techniques are almost always used in combination with others: for example Yang et al. [45] studied the adsorption mechanisms of Cr(VI) by a LDH intercalated with EDTA through a multi-approaches analysis which involved XRD, FTIR, X-ray photoelectron spectroscopy (XPS), and zeta potential analysis.

The standard co-precipitation method for LDHs synthesis, at first employed by Miyata [46], leads to a rigid spheroidal “sand rose” morphology of intergrown platelets. This kind of structure prevents accessibility to both the interlayers and external surface and produces less reactive LDHs. In this synthesis the precipitation of hydroxide particles starts from the beginning of the reaction, then nucleation and particle growth overlap resulting in a broad distribution of particle size.

Therefore, recently, increasing attention has been devoted toward the ability to produce LDHs displaying specific and unusual morphologies, which could increase the performance of the materials [47].

The presence of organic additives or mixed solvents during the synthesis by coprecipitation appeared as an efficient way to modify the LDH morphology. For example, the addition of biopolymers such as alginate has proved to strongly influence the LDH particle growth and, consequently, its morphology [48]. In fact, Wang et al. were able to produce three types of nanostructures (namely pompon-like, marigold-like, and coral-like LDHs) by simply adjusting the synthesis parameters including hydrothermal ageing time, concentration of metal ions, and reaction temperature.

**Figure 1:**
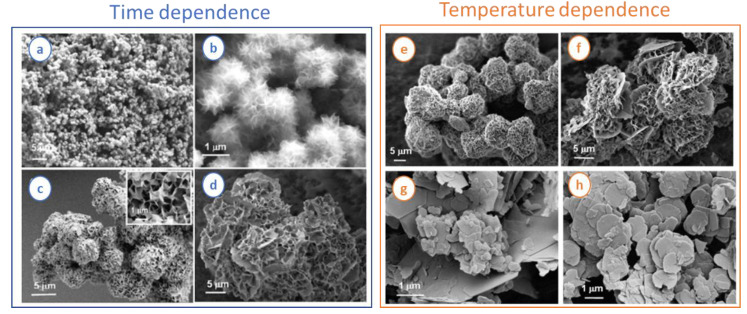
Field emission–scanning electron microscopy (FE-SEM) images of Ni/Al-CO_3_ layered double hydroxide (LDH). Samples a-d were prepared at 120 °C with different reaction times. (**a**,**b**) 1 h: LDH exhibits pompon-like morphology with diameter less than 1 μm. (**c**) 3 h: structure evolves into larger spherical sponge-like structures. (**d**) 12 h: flower-like morphology. Samples (**e**–**h**) were prepared with the fixed reaction time of 24 h but at (**e**) 80 °C, (**f**) 100 °C, (**g**) 150 °C, and (**h**) 180 °C. In this case, it is possible to observe sponge-like microspheres at low temperatures (60−80 °C) to large polydisperse stacked particles (200 nm−3 μm) at higher temperature (>150 °C). Adapted with permission from reference [49]. Copyright (2009), American Chemical Society.

A different way to prepare Ni/Al LDH nanoparticles was later proposed by Prevot et al. [49], and involved the use of a Ni glycinate complex as molecular precursor. The presence of this complex induced a slowdown of the LDH precipitation rate. As a result, the morphology depended on the synthesis time and the temperature used in the hydrothermal treatment (as can be seen in Figure 1). Throughout the reaction, particles went through a “pompon-like” intermediate phase which was further transformed into flower-like morphology. In this latter nanostructure, interconnected particles were associated on large thin disk-shaped particles. Moreover, the as-synthesized LDH flower-like morphology could be calcinated to produce mixed oxides displaying the same nanotextured morphology. LDH nanoparticles can be subsequently submitted to spray-drying to easily produce LDH microspheres which are of interest in many practical applications such as catalysis, chromatography, and sensors as well as controlled drug release [50].

Other strategies employ a support as sacrificial template which leads to porous LDH materials. For instance, LDH hollow capsules and 3D ordered macroporous materials have been prepared [51,52,53]. Yu et al. [54] fabricated hierarchical hollow nanoprisms based on ultrathin Ni/Fe LDH with enhanced OER performances through a facile self-templated strategy.

**Figure 2:**
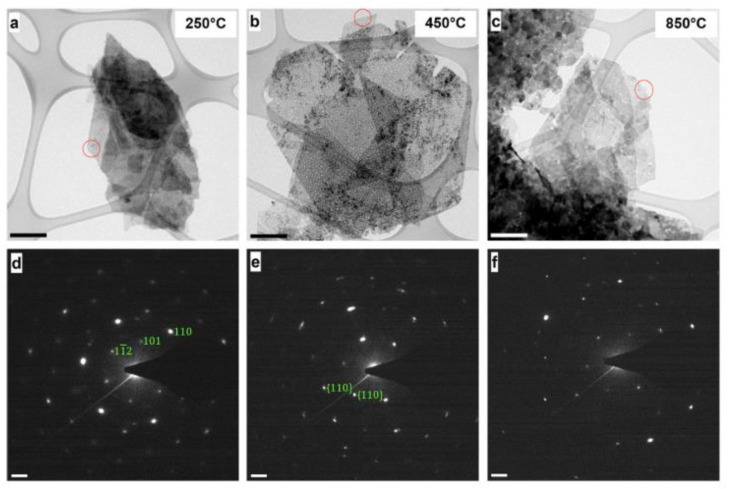
Transmission electron microscopy (TEM) images and associated selected area electron diffraction (SAED patterns of the Ni/Fe LDH. (**a**–**c**) TEM and (**d**–**f**) SAED patterns corresponding to the end of the 250 °C, 450 °C, and 850 °C steps of the applied heating ramp, respectively. Green annotations (**d**,**e**) represent labelled LDH crystallographic planes. Scale bars for TEM micrographs (**a**–**c**) and SAED patterns (**d**–**f**) are 200 nm and 2 nm^−1^. Figure adapted and taken from ref. [55]. Permissible to use under a CC-BY 4.0 license.

A commonly employed way to characterize nanosized materials is to couple transmission electron microscopy (TEM) and selected area electron diffraction (SAED) analysis. As an example, Hobbs et al. [55] applied in situ TEM to extensively characterize two LDH nanomaterials. The combined approach of TEM and SAED allowed both a morphological and crystallographic understanding of the systems (Figure 2). In particular, the study reported the investigation and comparison of the LDHs thermal degradation behaviors. Heating the Ni/Fe nanomaterials in situ, an amorphization was recorded at 250 °C, followed by a transition to a complex structure which could be described as NiO particles embedded in a NiFe_2_O_4_ matrix at 850 °C, as depicted in the figure. These observations were verified by high resolution and scanning TEM. Further electron microscopy characterization methodologies were also used, such as energy-filtered TEM, in order to observe directly the mechanistic behaviors in real time, and showed the evolution and nucleation to an array of spherical NiO nanoparticles on the platelet surfaces. The versatility of this approach was also confirmed by applying ex situ and in situ TEM and SAED characterization to a Mg/Al LDH structure which displayed an analogous behavior. The analysis was even paralleled with XRD, and identical phase transformations were verified, so demonstrating that the observed behaviors by TEM were due to the inherent properties of the LDH materials. The applicability of these methodologies established a suitable approach to characterize LDHs at a nanoscale level.

A different approach was followed by Dionigi et al. [56] which combined operando X-ray scattering and absorption spectroscopies to elucidate the active phase and the mechanism for the OER application. In spite of previous reports on the ex situ crystal structure of the as–synthesized precursors of M/Fe LDH catalysts and in situ local structure based on XAS measurements, little was previously known about the long–range crystal structures of the catalytically active phase under OER conditions. Combining electrochemical measurements with operando wide–angle X-ray scattering (WAXS) and XAS data, as well as ab initio molecular dynamic simulations, and a synergistic DFT approach that was benchmarked specifically for the strongly correlated Fe, Co, and Ni oxides and (oxy)hydroxides, the authors were able to unravel the crystal structures and electrocatalytic OER mechanisms of the active phases of Ni/Fe and Co/Fe LDH catalysts. They provided the first direct atomic–scale evidence that, under OER conditions, both Ni/Fe and Co/Fe LDHs transform from the as–prepared α–phase to a deprotonated γ–phase.

## 4. Applications

### 4.1. Oxygen Evolution Reaction (OER)

The production of an effective anode for OER is an enabling technology in the development of electrochemical devices for the conversion of electrical energy in chemical one and vice versa [57,58,59]. For example, the high cost of OER electrodes hinders large-scale hydrogen production by water electrolysis, and OER electrodes find applications in devices for CO_2_ reduction and air batteries [60,61,62]. The benchmark OER electrocatalysts are IrO_2_ and RuO_2_ as they are relatively stable at any pH in the operating conditions and offer high performance [61]. However, the development of catalysts for hydrogen evolution that efficiently work at high pH value has paved the way to the research concerning new classes of materials for OER [62]. Among them, LDHs are emerging materials due to their high performance and chemical tunability. The LDH structure allows for an enhancement of the catalytic features, since they display a high electroactive area and large availability of edge sites. When a layered structure is generated by adding a suitable trivalent cation to an OER electroactive hydroxide (or oxide) of a divalent cation, an increase of OER performances is observed [63,64].

LDHs mainly work in a strongly alkaline environment and the mechanism of the electrochemical process is still debated and depends on the catalyst composition and on the pH of the solution. The most commonly encountered reaction processes of LDH electrocatalysts in alkaline solution are listed below:

(1) $$\left[\mathrm{Me}\right]+{\mathrm{OH}}^{-}\to \left[\mathrm{Me}-\mathrm{OH}\right]+{\;e}^{-}$$

(2) $$\left[\mathrm{Me}-\mathrm{OH}\right]+{\mathrm{OH}}^{-}\to \left[\mathrm{Me}-O\right]+H_{2}O+{\;e}^{-}$$

(3) $$2\left[\mathrm{Me}-O\right]+{\mathrm{OH}}^{-}\to 2\left[\mathrm{Me}\right]+O_{2}$$

(4) $$\left[\mathrm{Me}-O\right]+{\mathrm{OH}}^{-}\to \left[\mathrm{Me}-\mathrm{OOH}\right]+{\;e}^{-}$$

(5) $$\left[\mathrm{Me}-\mathrm{OOH}\right]+{\mathrm{OH}}^{-}\to \left[\mathrm{Me}-\mathrm{OO}\right]+H_{2}O+{\;e}^{-}$$

(6) $$2\left[\mathrm{Me}-\mathrm{OO}\right]\to 2\left[\mathrm{Me}\right]+2O_{2}$$

The performance of an electrocatalyst for OER is evaluated by quantifying the onset potential, the overpotential at a defined current density (e.g., 10 mA cm^−2^), Tafel slope in mV dec^−1^, mass and specific activities, turnover frequency (TOF), Faradaic efficiency, and the long-time stability tests. Table 1 is explanatory of these parameters.

Table 2 shows the performances in terms of Tafel plot and overpotential at 10 mA cm^−2^ of LDHs based catalysts more recently described in the literature. Among binary systems, Ni- and Fe-based LDHs guarantee the best performance. It is worth highlighting the key role of Fe^3+^ to improve the performance of electrocatalysts based on metal hydroxides or oxides. Iron, at the concentration of few parts per million, boosts the OER occurring at anodes chemically modified by Ni hydroxide [63,64]. Other bimetallic LDHs that display good activity for OER are those containing Co/Fe [4], Ni/Co [65], Ni/Mn [66], and Co/Cu [67].

However, the OER activity can be boosted by adding a third metal which could tune electronic structures and the physical-chemical features in order to increase the electrical conductivity, the electron transfer efficiency, and the adsorption of oxygenated species. Furthermore, the third metal could also increase the available catalytic sites if the synthesis leads to porous structures or thinner sheets, and allow the generation of high-valence metal centers. Many attempts have been made to incorporate Co into Ni/Fe LDHs with the aim to reduce the thickness of the nanosheets [68] and to introduce high valence metal centers [69]. The addition of V to Ni/Fe LDHs guarantees a lower energy barrier for the step going from OH* to O* and the presence of a higher number of active centers on the catalyst surface [70]. Cr exhibits oxidation states that can vary from +1 to +6, with Cr^3+^ and Cr^4+^ which are very common and stable [71]. Cr doping can engender stronger electron interactions within the metal hydroxide matrix and enhance the synergy among metals so improving the OER performance of LDHs.

The anions intercalated inside the LDHs have aroused less attention than the layers metals composition. Zhou et al. thoroughly studied the effects of interlayer anions with different standard redox potential (E°) on OER performance of Ni/Al LDH nanosheets. The authors showed that the anion can change the OER onset potential value and the difference between the best performing system and the worst one can be about 150 mV [72]. In particular, the values of the onset potentials are linearly correlated with the E° values of the intercalated anions, because the anion can stabilize the metal high valence states that promote OER. Unfortunately, the intercalation of CO_3_^2−^ leads to a decrease in OER activity, so hindering the use of LDHs intercalated in air [73].

The main weak points of LDHs as OER catalysts are related to the low electrical conductivity and the low accessibility to the catalytic centers. The main route to overcome the former drawback is the combination of LDHs with carbon nanomaterials (graphene, reduced graphene oxide, and carbon nanotubes) [74,75,76,77], metal nanomaterials [78,79,80], or with other electrocatalytic active metal compounds (e.g., oxides, sulfides, selenides, phosphides, nitrides, borides, carbides, phosphates, and borates) to produce composite materials [64]. Furthermore, the presence of the second material promotes OER activity since it favors the adsorption of reaction intermediates, and stabilizes the high valence states of the active metal center thanks to the donation of negative charge. To address the latter drawback the exfoliation of LDH structures to obtain single sheets can ease the accessibility to active sites, and leads to theoretically specific areas higher than 1000 m^2^ g^−1^ [64]. For this reason, single layer LDHs show better OER performance than their bulk counterparts [81,82,83].

**Table 2:** Performances of LDHs based electrocatalysts for OER.

Catalyst	Conditions	Tafel Slope (mV dec^−1^)	Overpotential (mV) at 10 mA cm^−2^
Ni/Al [4]	1 M NaOH	29	370 *
Ni/Fe [4]	1 M NaOH	25	320 *
Co/Al [4]	1 M NaOH	30	370 *
Co/Fe [4]	1 M NaOH	29	310 *
NiCo [65]	0.1 M KOH	113	290 *
NiMn [66]	1 M KOH	30	220
CuCo [67]	1 M KOH	47	300
NiCoFe [69]	1 M KOH	48	232
NiFeV [70]	1 M KOH	42	192
NiFeCr [71]	1 M KOH	69	280
NiFe-CO_3_^2−^ [73]	1 M KOH	50	341
NiFe-Cl^−^ [73]	1 M KOH	47	343
NiFe on nanofiber [74]	1 M NaOH	21	260
NiFe-C [77]	1 M KOH	35	210
Cu@NiFe [78]	1 M KOH	28	199
Au/NiFe [79]	1 M KOH	36	237
Ni nanoparticle/NiFe [80]	1 M KOH	62	328
NiCo nanosheet [82]	1 M KOH	40	367
CoAl single layer	1 M KOH	36	252

* onset potential.

### 4.2. Other Energy Applications

Despite LDHs displaying the best performance when are used as electrocatalysts for OER, these materials have been successfully employed for other applications in the field of energy. Some papers describe LDHs as active materials in hydrogen evolution reaction (HER) occurring in the basic environment. In addition, further efforts have been devoted to the development and design of bi-functional catalysts for overall water splitting, which are able to catalyze both OER and HER when a suitable potential is applied. Ni/Fe LDH activity for HER is extremely poor in alkaline solutions (overpotential >210 mV at 10 mA cm^−2^), which results in a very high overall water-splitting voltage [84]. However, its performance can be boosted by doping with Ir [85] or Ru [86]. Since these metals low down the energy barriers in HER, the overpotential at 10 mA cm^−2^ is about 30 mV, which is a low value for the development of devices operating in the real world.

Since LDHs can act as HER and OER catalysts, they have been also proposed as bi-functional electrocatalysts for overall water splitting. Rajeshkhanna et al. introduced a Fe trace level doping of Ni and Co LDHs, pointing out an improved performance in the best operating conditions [87]. Liu et al. described ultrathin Co/Fe LDHs for electrochemical overall water splitting, highlighting the key role played by defects in the catalytic processes [88]. Although pure LDHs have been reported in the literature, the bi-functional electrocatalysts for overall water splitting are usually prepared by combining LDHs with other materials, such as graphitic carbon nitride [89,90], defective graphene [91], Cu nanowires [78], Ni nanotubes [87], and selenides [92,93]. The LDH deposition on electrode surface can be performed to obtain a well-organized nanostructure that boosts the diffusion of both reagents and products of the reaction [78,87]. The different phases inside the composite materials usually generate a synergic effect that leads to a higher performance than the single component alone. The high number of defects and typology of the active sites together with the ability to adsorb reagents and intermediates are at the basis of the success of these composite materials. Table 3 reports the performances of some LDHs and bi-functional materials for the water splitting reaction, as published in the last few years.

Finally, LDH has been proposed as an anode in fuel cells, thanks to the electrochemical ability to oxidize different substrates such as methanol [94,95,96], glucose [97], and urea [98,99,100]. LDHs act as mediators and extract electrons from the substrates, which are forced to reach the cathode and generate the electrical power. The oxidation would not take place at bare electrodes. The electrocatalytic process was studied by cyclic voltammetry, as described in the section “sensors”. The production of composite materials is also exploited in the field of fuel cells. To boost electrocatalysis, LDHs are combined with metal nanoparticles [83,96,98], which are preferred to carbon nanomaterials as they can also favor the oxidation of the substrate. In addition, LDHs stabilize the metal nanoparticles because they hinder their aggregation and increase the lifetime of the anode. Finally, synergic effects can occur during electrocatalysis, since the nanoparticles and LDHs can adsorb different reaction intermediates.

### 4.3. Sensors

Electrocatalysis mediated by LDHs has been exploited also to develop sensors. Beyond the strong points already mentioned in the previous paragraphs, when LDHs are used in the production of analytical tools their ion-exchange capability allows for an analyte pre-concentration that boosts performance [101].

**Figure 3:**
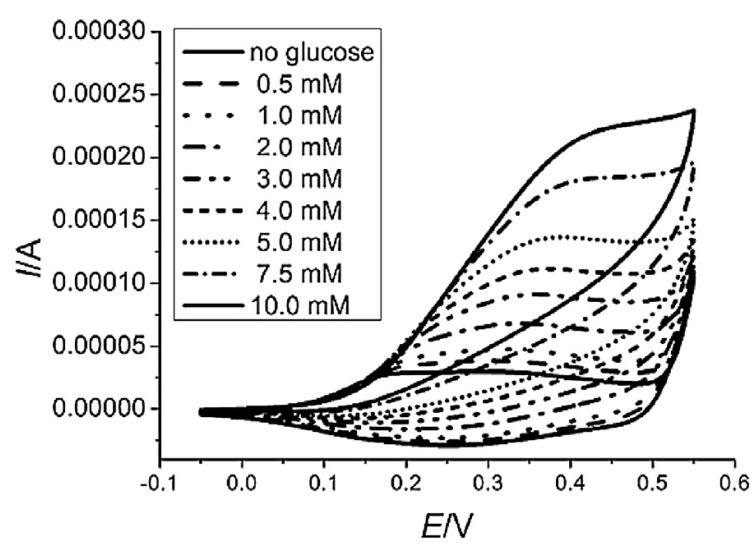
Cyclic voltammograms recorded at Pt modified with a Co/Al LDH in 0.1 M NaOH, containing glucose at different concentrations. Reprinted from ref. [117], Copyright (2015), with permission from Elsevier.

Tonelli’s group firstly investigated electrodes modified with a Ni/Al LDH for the detection of alcohols [102] which were applied also for real sample analysis [103]. Following these pivotal studies, the electrocatalytic features of Ni/Al, Co/Al, and Cu/Al LDHs have been exploited to develop sensors for the detection of sugars [104,105,106,107,108], amines [109,110], pesticides [111], chemicals [35,112], and phenols [110,113,114,115]. Due to the importance of a daily determination of blood sugar level for diabetics, several papers have proposed enzyme-less glucose sensors, based on Ni/Al LDHs modifying different substrates [105,106,109,116]. The oxidation of the above cited analytes does not occur at common bare electrodes, such as Pt, glassy carbon, or Au, due to the high overpotentials, but they become easily detectable thanks to the intervention of redox mediators such as LDHs. The transduction of the chemical signal in a current is due to an electrocatalytic mechanism that can be represented by the following reactions:

(7) $$\left[{\mathrm{Me}}_{\mathrm{red}}\right]-\mathrm{LDH}\;+{\;\mathrm{OH}}^{-}\;\to \;\left[{\mathrm{Me}}_{\mathrm{ox}}-\mathrm{OH}\right]-\mathrm{LDH}\;+{\;e}^{-}$$

(8) $$n\left[{\mathrm{Me}}_{\mathrm{ox}}-\mathrm{OH}\right]-\mathrm{LDH}\;+\;\mathrm{reduced}\;\mathrm{analyte}\;\to \;n\left[{\mathrm{Me}}_{\mathrm{red}}\right]-\mathrm{LDH}\;+\;\mathrm{oxidazed}\;\mathrm{analyte}$$

where Me_red_ LDH and Me_ox_–OH LDH represent the redox-active center in the reduced and oxidized states, respectively. Considering the bivalent cation as the redox-active species, e.g., Ni, the couple Ni^3+^/Ni^2+^ is involved in the process, and during the application of the anodic potential Ni^3+^ is produced with a consequent insertion of the hydroxide anion inside the structure to balance the excess of positive charge (reaction (7)). Therefore, a high pH is necessary for the reaction to occur. When the analyte comes into close contact with the Ni^3+^ centers, its oxidation takes place restoring the reduced LDH form, i.e., Ni^2+^, and consequently the hydroxide anions leave the LDH structure (not evidenced in reaction (8)). The electrocatalytic processes occurring at these modified electrodes can be highlighted by cyclic voltammetry studies. As an example, Figure 3 shows the CVs recorded for the oxidation of glucose at a Co/Al LDH modified electrode [117]. The redox waves associated with the LDH are clearly visible in glucose absence. Increasing the glucose concentration, the anodic peak current increases while the cathodic process, which is related to the presence of Me_ox_–OH LDH centers, disappears since they are completely consumed by the reaction with the analyte. This is the expected behavior of an efficient electrocatalytic process. The analyte detection can be carried out by cyclic voltammetry, chronoamperometry, and differential pulse voltammetry, depending on the concentration level that should be estimated. The performances of these devices are generally evaluated determining sensitivity, limit of detection (LoD), range of linearity, response time, repeatability/reproducibility, and lifetime (see Table 4).

When LDHs work as sensing material in electrochemical sensors, they exhibit the same weak points, such as low electrical conductivity and difficult access to catalytic sites, already pointed out for OER. Therefore, the same strategies can be employed to enhance the sensors’ performance. Composite materials based on LDHs and carbon nanomaterials have been synthesized to fabricate sensors for enzyme-less detection of glucose [34,118,119], guanine [120], H_2_S [121], and dopamine [122] displaying superior performance thanks to the enhanced electrical conductivity. The sensor performances can be improved increasing the accessibility to the redox-active sites, if the conductive support is chemically modified with ultrathin LDH layers [123,124,125]. Finally, high sensitivities can be reached using supports, such as carbon cloth [116,126] and carbon fiber [123], that improve the mass transport due to a high surface and/or porosity of their structure.

In Table 5, the performances of the sensors based on LDHs or their composites are shown, reporting the active material and the conductive support when it helps to increase the sensitivity of the detection. It can be noted that, depending on the analyte and the employed technique, the LoD can be very low, i.e., in the nM range.

## 5. Conclusions and Future Perspectives

Layered double hydroxides are promising materials in the field of electrocatalysis due to the high surface area, low cost, ion-exchange capability, and easy preparation, as demonstrated by their use in oxygen evolution reactions, electrochemical water splitting, fuel cells, sensors, etc. Peculiar functionalities can be obtained by tuning chemical composition. For example, the presence of a cation that can undergo reversible redox processes is mandatory in such applications because it acts as a redox mediator and provides sufficient electrical conductivity to the material by promoting electron hopping. The LDH composition should also be designed bearing in mind the final application. For example, the oxygen evolution reaction is eased by Fe^3+^ that is always present in OER catalysts with high performances. On the other hand, the solvent discharge must be avoided in sensing because it narrows the potential range wherein the devices can operate and detect the analytes. Thereby, Al^3+^ is preferred to Fe^3+^ as trivalent cation when developing electrochemical sensors that work in an aqueous environment. The chemical composition can be also varied by adding additional metal cations to obtain tri-metallic or multi-metallic LDHs wherein synergic effects can take place with the aim to stabilize the high oxidation state of the catalytic centers or the reaction intermediates. Moreover, the insertion of particular cations like Ir or Ru in the LDHs structure paves the way to catalyze other reactions, such as the hydrogen evolution. Although some papers also describe the role of the intercalated anions, this field appears unexplored.

The relatively low electrical conductivity, performance decay, site accessibility, and the lack of specific electrocatalytic activity can hamper the LDHs’ use in real applications. Therefore, recent literature has proposed some solutions to overcome these constraints. The synthesis of composites based on LDHs and carbon nanomaterials allows for the electrical conductivity to be boosted, while the combination with metal nanomaterials or selenides enhances the catalytic activity in the hydrogen evolution reaction for application in water splitting. Furthermore, the LDHs exfoliation is a synthetic route which enables the production of electrocatalysts with a large surface area and high accessibility to the active centers. In conclusion, LDHs-based catalysts benefit fundamentally from their versatility in terms of the flexibility of structure and composition, low cost, and highly efficient performances in a variety of reactions. The possibility for LDHs to be used in practical industrial-scale applications is close.

## Figures and Tables

**Table 1:** Main parameters to evaluate the oxygen evolution reaction (OER) performance of an electrocatalyst.

**Onset potential**	The potential at which the current starts to increase due to the occurrence of water oxidation.
**Overpotential at 10 mA cm^−2^**	The difference between the potential at which a current density of 10 mA cm^−2^ is recorded and the thermodynamic potential for O_2_ + 4e^−^ + 4 H^+^ ⇄ 2H_2_O
**Mass and specific activities**	Current densities divided by the catalyst loading (expressed as mass or volume)
**Turnover frequency (TOF)**	The moles number of O_2_ generated per time unit at a defined overpotential
**Faradaic efficiency**	The percentage of charge that flows at the electrode, and is used to produce O_2_
**Long-term stability test**	Long-time experiment under OER conditions to verify the mantainance of performance

**Table 3:** Performances of LDHs and bi-functional materials for the water splitting reaction.

Catalyst	Conditions	Voltage * (V)	OER		HER	
			Overpotential (mV) at 10 mA cm^−2^	Tafel Slope (mV dec^−1^)	Overpotential (mV) at 10 mA cm^−2^	Tafel Slope (mV dec^−1^)
Cu nanowires NiFe [78]	1 M KOH	1.54	199	28	116	59
Ir^4+^ doped NiFe [85]	1 M KOH	1.41	200	-	34	32
NiFe [84]	1 M NaOH	1.70	240	-	210	-
NiFeRu [86]	1 M KOH	1.52	225	-	29	31
Ni nanotubes NiFe [87]	1 M KOH	1.51	191	41	101	101
Defective CoFe [88]	1 M KOH	1.63	300	40	255	95
CoFe C_3_N_4_ [89]	1 M KOH	1.82	275	58	417	77
Co_0.4_Fe_0.6_/g-CN_x_ [90]	1 M KOH	1.61	280	29	270	79
CoSe/NiFe [92]	1 M KOH	1.53	201	39	98	89
NiFe/NiSe [93]	1 M KOH	1.53	240 **	66	270 **	70

* Voltage of symmetric electrochemical cell for water splitting operating at 10 mA cm^−2^; ** measured at 100 mA cm^−2^.

**Table 4:** Main parameters used to evaluate the performance of an electrochemical sensor.

**Sensitivity**	The slope of the calibration line (the first derivative for a curve). The calibration line is obtained by plotting the current vs. the analyte concentrations of standard solutions.
**Limit of detection**	The lowest quantity of the analyte that can be distinguished from the blank (absence of analyte) at a fixed statistical confidence level. It is the analyte concentration (or amount) that generates a signal equal to the blank signal plus n times its standard deviation (usually 3).
**Range of linearity**	The concentration range wherein the response can be approximated by a line. The lower limit is usually the limit of detection (LoD) value. The upper limit is the concentration value at which the calibration curve departs from linearity (limit of linearity; LoL).
**Response time**	The time required to reach 90% of the signal. It is evaluated by measuring the signal after a variation of the concentration.
**Repeatability/Reproducibility**	Evaluated repeating the same measurements with the same sensor/different sensors, and usually expressed as standard deviation.
**Lifetime**	Evaluated by analyzing the same solution or acquiring calibration lines over time, usually for weeks or months. It is the time after which the signal is decreased by a fixed value (for example 10%).

**Table 5:** Analytes detected by sensors based on LDHs or their composites.

Electrode Modifier	Analyte	Tecnhique	LoD	Conditions
NiAl [102]	Methanol, Ethanol	CV	3 ppm	0.1 M NaOH
NiAl [103]	Ethanol	CV	5 mM	0.1 M NaOH
Pt nanoparticles + NiAl [104]	Ethanol	A	0.05 mM	0.1 M NaOH
	Glucose	A	0.025 mM	0.1 M NaOH
NiAl [105]	Glucose	CV	-	0.1 M NaOH
NiAl + chitosane [106]	Glucose	CA	0.01 mM	0.1 M NaOH
CuAl [107]	Glucose	A	0.02 µM	0.1 M NaOH
NiAl CNT [108]	Glucose	CV	-	0.1 M NaOH
NiAl [109]	Alphatic ammines	A	0.7 µM	0.1 M NaOH
	Aromatic ammines	A	6 µM	0.1 M NaOH
NiAl [110]	Phenol	A	1 µM	0.1 M NaOH
	Glucose	A	0.01 mM	0.1 M NaOH
NiAl [111]	Glyphosate	A	1 µM	0.1 M NaOH
	Glufosinate	A	5 µM	0.1 M NaOH
NiFe [112]	H_2_O_2_	A	0.5 µM	0.1 M NaOH
CoAl [113]	Salycilyc Acid	A	0.2 µM	0.1 M NaOH
CoAl [114]	Aniline	A	0.02 µM	0.1 M NaOH
	Phenol	A	0.3 µM	0.1 M NaOH
NiAl [115]	Bisphenol A	DPV	7 nM	0.1 M pH 8.5 phosphate buffer solution
NiAl on carbon cloth [116]	Glucose	A	0.2 µM	0.1 M NaOH
CoAl [117]	Glucose (+ other sugar)	A	10 µM	0.1 M NaOH
CNF@NiCo [118]	Glucose	A	0.03 µM	0.1 M NaOH
NiAl/Electrochemical reduced Graphene Oxide [34]	Glucose	A	0.6 µM	0.1 M NaOH
NiAl/GO [120]	Guanine	LSV	3 nM	0.1 M pH 7 posphate buffer solution
	Adenine	LSV	20 nM	0.1 M pH 7 posphate buffer solution
CNTs@CuMn [121]	H_2_S	A	0.3 nM	0.1 M Posphate buffer saline
NiAl/graphene [122]	Dopamine	CV	0.1 mM	0.1 M NaOH
Ultrathin NiFe [123]	Glucose	A	0.6 µM	0.1 M NaOH
NiAl nanosheets [124]	Glucose	A	5 µM	0.1 M NaOH
CoAl/Naphthol Green B [125]	H_2_O_2_	A	0.9 µM	0.1 M NaOH
NiFe Carbon cloth [126]	NO_2_^−^	A	0.02 µM	Posphate Buffer Saline

A, amperometry; CV, cyclic voltammetry; DPV, differential pulse voltammetry; LSV, linear sweep voltammetry.
